# Supplementary material for: A Critical View of the Application of the APEX Software (Aqueous Photochemistry of Environmentally-Occurring Xenobiotics) to Predict Photoreaction Kinetics in Surface Freshwaters
Source: Molecules. 2019 Dec 18;25(1):9. doi: 10.3390/molecules25010009 (PMC7017383; doi:10.3390/molecules25010009)
Supplement: Supplementary file 1 [file molecules-25-00009-s001.zip › 3.2.4_gcc-4.4.0.zip!/3.2.4_gcc-4.4.0/3.2.4_gcc-4.4.0/doc/octave/html/interpreter/Patch-Properties.html]

Patch Properties - Untitled


Next: Surface Properties,
Previous: Image Properties,
Up: Graphics Object Properties


---

##### 15.2.2.7 Patch Properties

`cdata` `xdata` `ydata` `zdata`: Data defining the patch object. `facecolor`: The fill color of the patch. See Colors. `facealpha`: A number in the range [0, 1] indicating the transparency of the patch. `edgecolor`: The color of the line defining the patch. See Colors. `linestyle` `linewidth`: See Line Styles. `marker` `markeredgecolor` `markerfacecolor` `markersize`: See Marker Styles.
